# Supplementary material for: Beyond oscillations—Toward a richer characterization of brain states
Source: Imaging Neurosci (Camb). 2025 Feb 27;3:imag_a_00499. doi: 10.1162/imag_a_00499 (PMC12320016; doi:10.1162/imag_a_00499)
Supplement: Supplementary Material [file imag_a_00499-supp.pdf]

Supporting information

Divergence from pre-registration (LEMON data)

The pre-registration stated that on top of TimeFeats, fullFFT and FreqBands, a fourth model, namely First Two Moments (FTM) would also have been added to the comparison. This model, as the name suggests, included the first two moments of a probability distribution, namely mean and standard deviation, as features. The importance, as well as the explanatory power of these two features has been long underestimated, as highlighted recently (Henderson, Bryant, & Fulcher, 2023).
When we computed the classification accuracy for the FTM model, although it was still remarkably high, it was still strongly outperformed from the other models (Figure S1). In order to make our story easier for the reader, we decided to remove FTM from the results altogether.

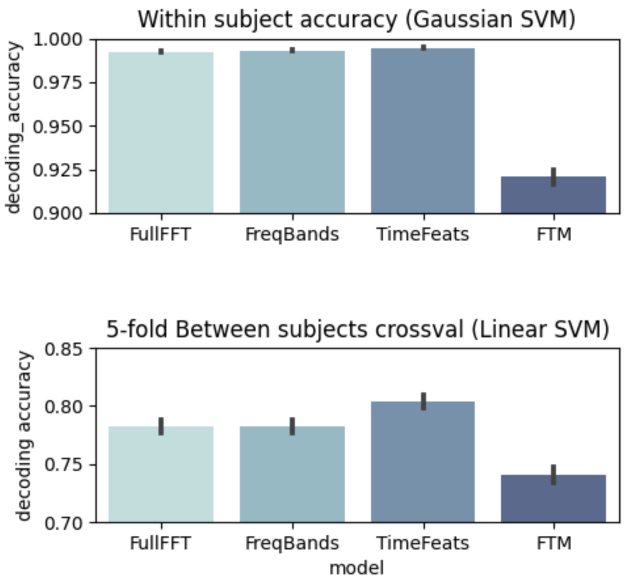

Figure S1: extended results for the classification of EC/EO on the replication dataset including the FTM.

Comparison of different methods for extracting aperiodic components from the spectra

Since the estimation of aperiodic components of the spectra relied on a custom method (see Methods), we compared it, in the within subjects classification, to the more established FOOOF algorithm (Figure S2).

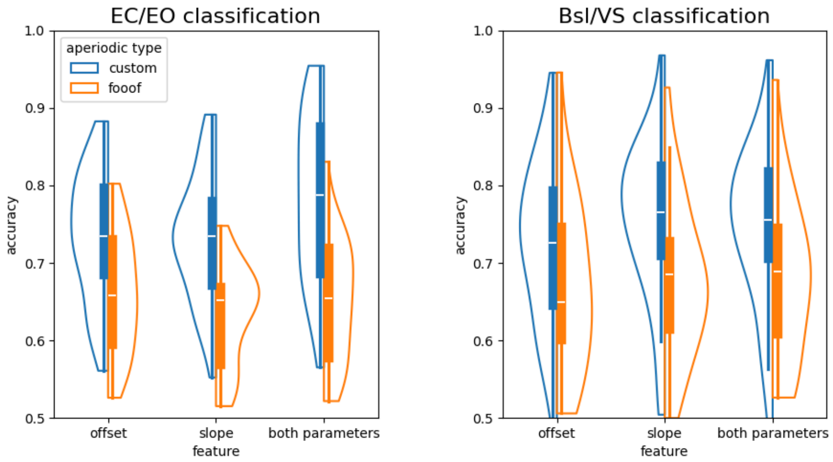

Figure S2: Comparison brain states classification accuracy from different algorithms (fooof vs custom) for the extraction of aperiodic components from the spectra. Classification accuracy is estimated within subjects.

In general, the custom method obtained higher accuracies than the FOOOF estimated at the single trial level. One plausible explanation for this advantage might come from the fact that the custom method capitalizes on the removal of oscillatory components which are estimated on the average of all trials, and thus yielding a more precise estimate of oscillatory components than the one performed at the single trial level.
Nonetheless, aperiodic components were widely outperformed by both oscillatory and non-oscillatory features. Moreover, the implementation of the FOOOF against the custom method did not change anyhow the overall pattern of the results on the aggregated features. For this reason we opted, for sake of consistency across the paper, to use the custom method,

Comparison of different classifiers

In the main paper all classification accuracies were estimated using Support Vector Machine (SVM), as it is widely adopted in neuroscience (Subasi & Ismail Gursoy, 2010); (Grootswagers, Wardle, & Carlson, 2017); (Guggenmos, Sterzer, & Cichy, 2018; Horikawa, Tamaki, Miyawaki, & Kamitani, 2013). For sake of completeness, we also tested classification accuracy also with other classifiers, namely Linear Discriminant Analysis (LDA) and Logistic Regression (see Figure S3).

All three classifiers confirmed the pattern of higher accuracy for TimeFeats, with SVM performing better than the other two. This result consolidates the central finding of the paper, namely that features of the TimFeats lead to higher classification accuracy of the brain states examined. Concurrently this result reaffirms SVM as the best classifier choice among the options considered.

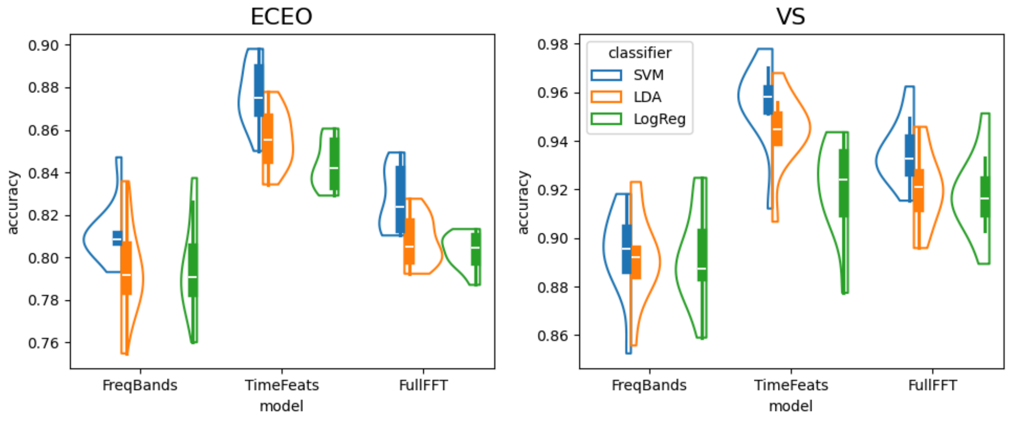

Figure S3: Comparison of brain states classification accuracy from different classifiers.

Alternatives to accuracy as metric for informativeness

Balanced accuracy, the metric used throughout the paper, is a valid way to evaluate performance of models in classification tasks. Nonetheless there are other ways that can be quite expressive for the evaluation of features informativeness, such as by examining the weights assigned by the classifiers, provided that the classifier does not enforce a nonlinear relationship between the variables. For this reason, we fitted an additional linear SVM on the across subjects data, this time merging together the TimeFeats and fullFFT in the feature space. If the features contained in the TimeFeats set are actually more informative they should show higher coefficients (figure S4).
As expected the highest coefficient values are contained in the TimeFeats set, suggesting their strong importance, and hence informativeness, in the classification of brain states.

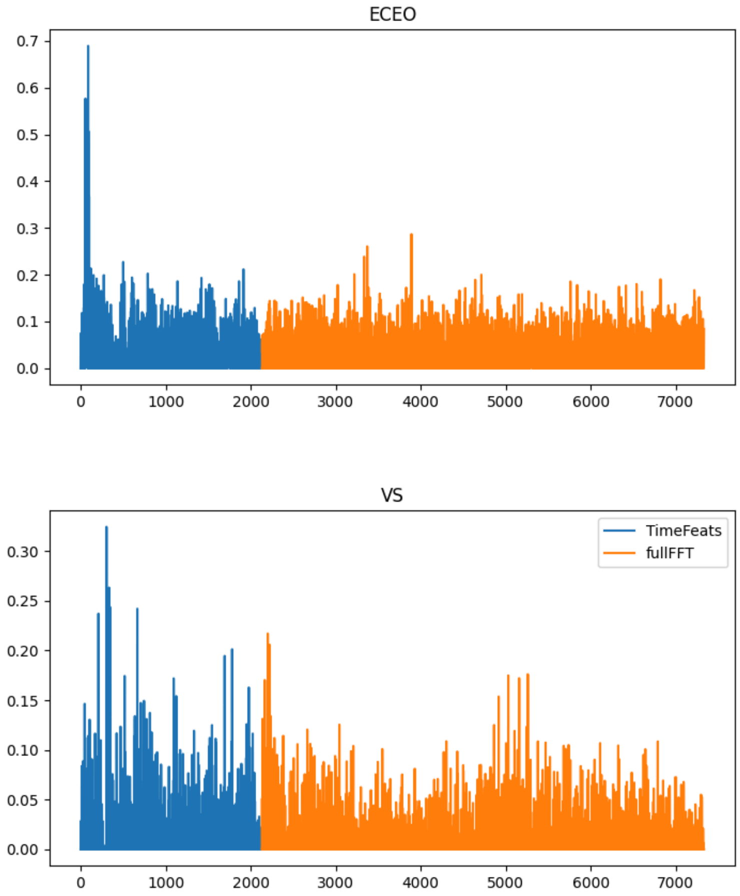

Figure S4: weights assigned to single features from the linear SVM in the classification of different states. Each combination of feature-parcel is represented as a bar on the X axis, with color codes for the TimeFeats vs FullFFT. The Y axis shows the absolute value of the weight assigned by the classifier. The upper inset indicates the weights for the EC/EO classification, the lower inset the classification between bsl/VS.

Effect of normalization of time series before feature extraction

“Simple” features characterizing the distribution of time series, like mean and standard deviation (std), resulted often among the top ranking ones. Another interesting perspective, more oriented to the dynamics of the time series rather than their own distribution is obtained by normalizing each time series before extracting features. This re-analysis (Figure S5) confirms the Timefeats set as the best performer overall in both classifications. For the VS/BSL classification, the first single feature is high gamma power, somewhat congruent with our previous results on the 4 labels classification task that pinpointed high gamma power as the most informative frequency band among the ones considered. The other 3 features from TimeFeats closely following -and almost identical in accuracy to- high gamma power are all related to the autocorrelation function (AF), as the first 1/e crossing of AF, the first minimum of AF, and one metric of automutual information. This confirms once again the role of gamma power in

characterizing the transition between baseline and visual stimulation, while suggesting that this change in spectral power can also be well characterized by changes in the autocorrelation function.

Intriguingly, in the ECEO comparison none of the spectral-based features resulted in the top single features. The winning single features are instead describing distances in a 2-d space of time series (CO_Embed2) and their discrete symbolization (SB_MotifThree).

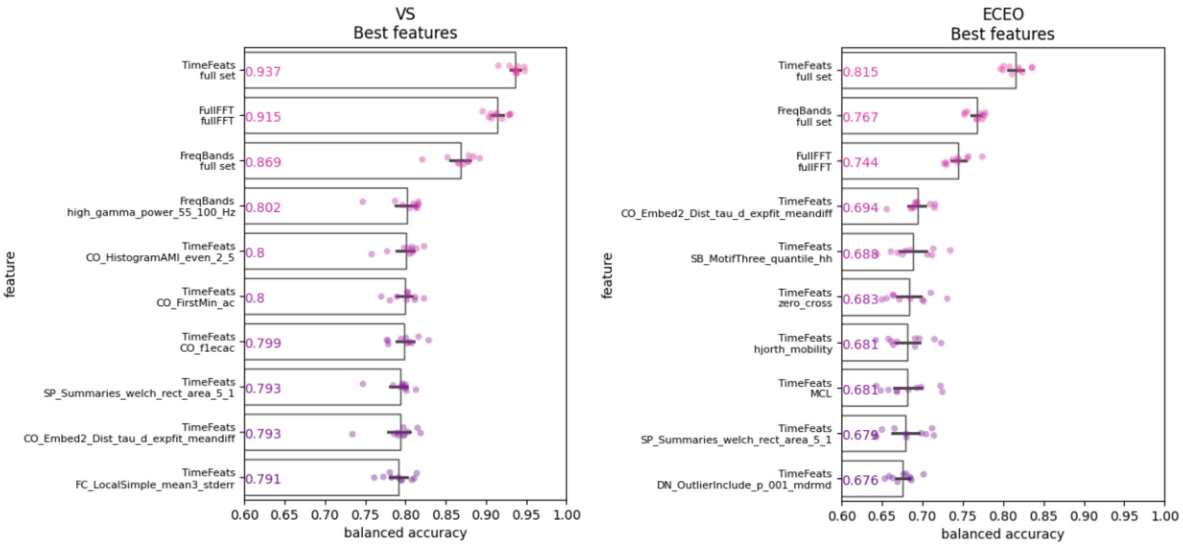

Figure S5: Classification accuracy across subjects for the top 10 features, including full sets, after normalization at the time series level. Left inset shows the classification of BSL/VS, whereas the right inset shows the classification between EC/EO.

source	feature name	feature explanation	feature set model
catch22 (Lubba et al. 2019)<br>reduced version of the bigger hctsa ensemble<br>(Fulcher et al. 2013; Fulcher and Jones 2017)	DN_HistogramMode_5	Mode of z-scored distribution (5-bin histogram)	TimeFeats
	DN_HistogramMode_10	Mode of z-scored distribution (10-bin histogram)	TimeFeats
	SB_BinaryStats_mean_longstretch1	Longest period of consecutive values above the mean	TimeFeats
	DN_OutlierInclude_p_001_mdrmd	Time intervals between successive extreme events above the mean	TimeFeats
	DN_OutlierInclude_n_001_mdrmd	Time intervals between successive extreme events below the mean	TimeFeats
	CO_f1ecac	First 1/e crossing of autocorrelation function	TimeFeats
	CO_FirstMin_ac	First minimum of autocorrelation function	TimeFeats
	SP_Summaries_welch_rect_area_5_1	Total power in lowest fifth of frequencies in the Fourier power spectrum	TimeFeats
	SP_Summaries_welch_rect_centroid	Centroid of the Fourier power spectrum	TimeFeats
	FC_LocalSimple_mean3_stderr	Mean error from a rolling 3-sample mean forecasting	TimeFeats
	CO_trev_1_num	Time-reversibility statistic, ((x_{t+1} − x_{t})^3)∣t	TimeFeats
	CO_HistogramAMI_even_2_5	Automutual information, m = 2, τ = 5	TimeFeats
	IN_AutoMutualInfoStats_40_gaussian_fmmi	First minimum of the automutual information function	TimeFeats
	MD_hrv_classic_pnn40	Proportion of successive differences exceeding 0.04σ (Mietus 2002)	TimeFeats
	SB_BinaryStats_diff_longstretch0	Longest period of successive incremental decreases	TimeFeats
	SB_MotifThree_quantile_hh	Shannon entropy of two successive letters in equiprobable 3-letter symbolization	TimeFeats
	FC_LocalSimple_mean1_tauresrat	Change in correlation length after iterative differencing	TimeFeats
	CO_Embed2_Dist_tau_d_expfit_meandiff	Exponential fit to successive distances in 2-d embedding space	TimeFeats
	SC_FluctAnal_2_dfa_50_1_2_logi_prop_r1	Proportion of slower timescale fluctuations that scale with DFA (50% sampling)	TimeFeats
	SC_FluctAnal_2_rsrangefit_50_1_logi_prop_r1	Proportion of slower timescale fluctuations that scale with linearly rescaled range fits	TimeFeats
	SB_TransitionMatrix_3ac_sumdiagcov	Trace of covariance of transition matrix between symbols in 3-letter alphabet	TimeFeats
	PD_PeriodicityWang_th0_01	Periodicity measure (Wang et al. 2007)	TimeFeats
hctsa (Fulcher et al. 2013; Fulcher and Jones 2017)	DN_FitKernelSmoothraw_entropy	Statistics of a kernel-smoothed distribution of the data	TimeFeats
	DN_FitKernelSmoothraw_max		TimeFeats
MATLAB built-in functions	mean		TimeFeats
	median		TimeFeats
	std	standard deviation	TimeFeats
	iqr	interquantile range	TimeFeats
	mad	mean absolute deviation	TimeFeats
	skewness		TimeFeats
	kurtosis		TimeFeats
custom or re-adapted	SAMPEN	SAMPle ENtropy (adapted MATLAB function from Kijoon Lee, 2012)	TimeFeats
	wpH	Wighted Permutation Entropy (Fadlallah et al. 2013; Waschke et al. 2017)	TimeFeats
	zero_cross	number of zero crossing points of the timeseries	TimeFeats
	zero_cross_derivative	number of zero crossing points of the timeseries' derivative	TimeFeats
	MCL	mean(abs(diff(SIGNAL)^2)). Similar implementation in Yahyaei and Özkurt 2022	TimeFeats
	hjorth_mobility	This is defined as the square root of variance of the first derivative of the signal y(t) divided by variance of the signal y(t).	TimeFeats
	hjorth_complexity	hjorth mobility(dy(t)/y(t)) / hjorth mobility(y(t))	TimeFeats
	aperiodic_slope	aperiodic components (offset and slope) of the FFT spectra (adapted from Donoughe et al. 2020)	TimeFeats
	aperiodic_offset		TimeFeats
	Hurst_exp	Hurst Exponent, as measure of long-term memory of time series.	TimeFeats
	FFT spectrum	FFT spectrum from 1 to 100 Hz (1 Hz resolution)	FullFFT
	delta_power	FFT power averaged in the delta band (1-4 Hz)	FreqBands
	theta_power	FFT power averaged in the theta band (4-8 Hz)	FreqBands
	alpha_power	FFT power averaged in the alpha band (8-13 Hz)	FreqBands
	beta_power	FFT power averaged in the beta band (13-30 Hz)	FreqBands
	low_gamma_power	FFT power averaged in the low gamma band (30-45 Hz)	FreqBands
	high_gamma_power	FFT power averaged in the high gamma band (55-100 Hz)	FreqBands

Table 1: full list of all the features used in the study

regionName	regionLongNam	regionIdLabel	LR	region	Lobe	cortex	regionID	Cortex_ID	x-cog	y-cog	z-cog	volmm
V1_R	Primary_Visual_C	201_R	R	V1	Occ	Primary_Visual	201	1	78.060375	44.539286	74.333474	7089
MST_R	Medial_Superior_	202_R	R	MST	Occ	MT+_Complex_and_Neighboring	202	5	43.620295	63.751227	78.013093	611
V6_R	Sixth_Visual_Are	203_R	R	V6	Occ	Dorsal_Stream_Visual	203	3	72.226868	49.256228	102.929715	1124
V2_R	Second_Visual_A	204_R	R	V2	Occ	Early_Visual	204	2	77.026083	47.558042	76.305265	6211
V3_R	Third_Visual_Are	205_R	R	V3	Occ	Early_Visual	205	2	71.976821	41.580308	78.859282	4875
V4_R	Fourth_Visual_Ar	206_R	R	V4	Occ	Early_Visual	206	2	57.656017	41.782393	71.003195	2817
V8_R	Eighth_Visual_Ar	207_R	R	V8	Occ	Ventral_Stream_Visual	207	4	60.698264	51.949266	59.301736	749
V3A_R	Area_V3A_R	213_R	R	V3A	Occ	Dorsal_Stream_Visual	213	3	75.553398	36.584466	102.458252	1545
V7_R	Seventh_Visual_	216_R	R	V7	Occ	Dorsal_Stream_Visual	216	3	62.285714	42.400534	103.668892	749
IPS1_R	IntraParietal_Sul	217_R	R	IPS1	Par	Dorsal_Stream_Visual	217	3	64.710065	55.195753	111.066482	1083
FFC_R	Fusiform_Face_C	218_R	R	FFC	Temp	Ventral_Stream_Visual	218	4	49.72947	75.126159	51.324834	3020
V3B_R	Area_V3B_R	219_R	R	V3B	Occ	Dorsal_Stream_Visual	219	3	60.919386	48.422265	91.56238	521
LO1_R	Area_Lateral_Oc	220_R	R	LO1	Occ	MT+_Complex_and_Neighboring	220	5	47.281977	45.175872	76.959302	688
LO2_R	Area_Lateral_Oc	221_R	R	LO2	Occ	MT+_Complex_and_Neighboring	221	5	44.07	44.375	67.918333	600
PIT_R	Posterior_InferoT	222_R	R	PIT	Occ	Ventral_Stream_Visual	222	4	44.641614	49.954905	58.057753	1264
MT_R	Middle_Tempora	223_R	R	MT	Occ	MT+_Complex_and_Neighboring	223	5	38.58547	57.830484	80.888889	702
PH_R	Area_PH_R	338_R	R	PH	Temp	MT+_Complex_and_Neighboring	338	5	41.367731	64.734587	61.13861	2287
V6A_R	Area_V6A_R	352_R	R	V6A	Par	Dorsal_Stream_Visual	352	3	66.971053	43.206579	117.961842	760
VMV1_R	VentroMedial_Vis	353_R	R	VMV1	Occ	Ventral_Stream_Visual	353	4	71.942782	73.198944	63.584507	1136
VMV3_R	VentroMedial_Vis	354_R	R	VMV3	Occ	Ventral_Stream_Visual	354	4	62.83642	64.92284	61.896605	648
V4t_R	Area_V4t_R	356_R	R	V4t	Occ	MT+_Complex_and_Neighboring	356	5	41.209816	53.52638	69.720245	815
FST_R	Area_FST_R	357_R	R	FST	Occ	MT+_Complex_and_Neighboring	357	5	38.91249	62.962609	72.511535	1257
V3CD_R	Area_V3CD_R	358_R	R	V3CD	Occ	MT+_Complex_and_Neighboring	358	5	51.141138	43.050328	83.778993	914
LO3_R	Area_Lateral_Oc	359_R	R	LO3	Par	MT+_Complex_and_Neighboring	359	5	41.930187	53.481809	85.433628	1017
VMV2_R	VentroMedial_Vis	360_R	R	VMV2	Occ	Ventral_Stream_Visual	360	4	62.482	72.616	65.532	500
VVC_R	Ventral_Visual_C	363_R	R	VVC	Temp	Ventral_Stream_Visual	363	4	61.276977	79.81999	52.749876	2011
V1_L	Primary_Visual_C	1_L	L	V1	Occ	Primary_Visual	1	1	100.491589	41.138901	71.63704	6717
MST_L	Medial_Superior_	2_L	L	MST	Occ	MT+_Complex_and_Neighboring	2	5	132.416667	58.901786	82.059524	336
V6_L	Sixth_Visual_Are	3_L	L	V6	Occ	Dorsal_Stream_Visual	3	3	104.543112	44.481665	103.916749	1009
V2_L	Second_Visual_A	4_L	L	V2	Occ	Early_Visual	4	2	102.236656	44.064791	74.401125	6220
V3_L	Third_Visual_Are	5_L	L	V3	Occ	Early_Visual	5	2	107.926111	40.632159	76.961153	4994
V4_L	Fourth_Visual_Ar	6_L	L	V4	Occ	Early_Visual	6	2	120.481621	41.344329	69.955702	3183
V8_L	Eighth_Visual_Ar	7_L	L	V8	Occ	Ventral_Stream_Visual	7	4	123.013193	51.8927	56.759015	1137
V3A_L	Area_V3A_L	13_L	L	V3A	Occ	Dorsal_Stream_Visual	13	3	104.528505	33.597072	99.335131	1298
V7_L	Seventh_Visual_	16_L	L	V7	Occ	Dorsal_Stream_Visual	16	3	114.850993	39.443709	100.271523	604
IPS1_L	IntraParietal_Sul	17_L	L	IPS1	Par	Dorsal_Stream_Visual	17	3	114.19184	51.537326	110.430556	1152
FFC_L	Fusiform_Face_C	18_L	L	FFC	Temp	Ventral_Stream_Visual	18	4	133.940136	69.78536	52.618797	3224
V3B_L	Area_V3B_L	19_L	L	V3B	Occ	Dorsal_Stream_Visual	19	3	117.755656	42.828054	88.434389	442
LO1_L	Area_Lateral_Oc	20_L	L	LO1	Occ	MT+_Complex_and_Neighboring	20	5	132.592541	39.361878	78.374309	724
LO2_L	Area_Lateral_Oc	21_L	L	LO2	Occ	MT+_Complex_and_Neighboring	21	5	137.691983	45.555907	68.914557	948
PIT_L	Posterior_InferoT	22_L	L	PIT	Occ	Ventral_Stream_Visual	22	4	137.394161	48.92805	61.153285	959
MT_L	Middle_Tempora	23_L	L	MT	Occ	MT+_Complex_and_Neighboring	23	5	133.489855	55.521739	82.907246	345
PH_L	Area_PH_L	138_L	L	PH	Temp	MT+_Complex_and_Neighboring	138	5	139.578644	61.953921	65.579087	2257
V6A_L	Area_V6A_L	152_L	L	V6A	Occ	Dorsal_Stream_Visual	152	3	112.534483	39.66092	114.188218	696
VMV1_L	VentroMedial_Vis	153_L	L	VMV1	Occ	Ventral_Stream_Visual	153	4	110.244105	74.319001	64.307906	721
VMV3_L	VentroMedial_Vis	154_L	L	VMV3	Occ	Ventral_Stream_Visual	154	4	119.618762	64.508982	61.017964	501
V4t_L	Area_V4t_L	156_L	L	V4t	Occ	MT+_Complex_and_Neighboring	156	5	138.695811	48.185792	74.724954	549
FST_L	Area_FST_L	157_L	L	FST	Occ	MT+_Complex_and_Neighboring	157	5	137.648765	57.787325	76.535983	931
V3CD_L	Area_V3CD_L	158_L	L	V3CD	Occ	MT+_Complex_and_Neighboring	158	5	125.152542	36.195686	81.429892	649
LO3_L	Area_Lateral_Oc	159_L	L	LO3	Occ	MT+_Complex_and_Neighboring	159	5	136.842415	48.964654	85.864507	679
VMV2_L	VentroMedial_Vis	160_L	L	VMV2	Occ	Ventral_Stream_Visual	160	4	119.360502	74.39185	66.366771	319
VVC_L	Ventral_Visual_C	163_L	L	VVC	Temp	Ventral_Stream_Visual	163	4	121.913003	74.920407	53.816752	2161

Table 2: full list of parcels selected in the MEG study

References

Grootswagers, T., Wardle, S. G., & Carlson, T. A. (2017). Decoding Dynamic Brain Patterns from Evoked Responses: A Tutorial on Multivariate Pattern Analysis Applied to Time Series Neuroimaging Data. *Journal of Cognitive Neuroscience*, *29*(4), 677–697.

Guggenmos, M., Sterzer, P., & Cichy, R. M. (2018). Multivariate pattern analysis for MEG: A comparison of dissimilarity measures. *Neuroimage*, *173*, 434–447.

Henderson, T., Bryant, A. G., & Fulcher, B. D. (2023). Never a Dull Moment: Distributional Properties as a Baseline for Time-Series Classification.

Horikawa, T., Tamaki, M., Miyawaki, Y., & Kamitani, Y. (2013). Neural decoding of visual imagery during sleep. *Science*, *340*(6132), 639–642.

Subasi, A., & Ismail Gursoy, M. (2010). EEG signal classification using PCA, ICA, LDA and support vector machines. *Expert systems with applications*, *37*(12), 8659–8666.
